# Positional Anchors and Cooperative Retained-Position Sets Define Non-Equivalent RNA Sequence Landscapes

**DOI:** 10.1007/s00239-026-10345-0

**Published:** 2026-09-11

**Authors:** Madhan R. Tirumalai, Rajat Rastogi, George E. Fox

**Affiliations:** 1 Department of Biology, Texas A&M University, TX 77843 College Station, USA; 2 Agentic World LLC, 1968 S. Coast Hwy, Suite 2124, Laguna Beach, CA 92651 USA; 3 Department of Biology and Biochemistry, University of Houston, Houston, TX 77204-5008 USA

**Keywords:** RNA sequence space, RNA secondary-structure prediction, Minimum free energy, Positional retention, Cooperative retained positions, Computational selection, Ribosomal peptidyl transferase center, ViennaRNA, G·U wobble pairing

## Abstract

Different RNA sequences do not have equal access to nearby structural sequence space, but how strongly local sequence neighborhoods depend on starting-sequence identity and individual nucleotide positions remains unclear. Here, we compared the fully mutated neighborhoods of three isolated 20-nucleotide RNA seeds inspired by the P-site peptidyl transferase center, shifted P-loop, and A-site A-loop regions of the ribosomal core using ViennaRNA minimum-free-energy secondary-structure predictions. The seeds were treated as conceptual starting points for sequence-space exploration rather than exact residue-numbered extracts from a single modern rRNA. The three seeds generated reproducibly different predicted foldability landscapes, with the shifted P-loop-inspired seed consistently producing the highest foldability across independent pools and increasingly stringent MFE thresholds. Single-position retention revealed strong positional effects: retained guanine generally increased predicted foldability, but one retained-G position in each seed significantly outperformed other retained-G positions within the same sequence. Disabling G·U wobble pairing reduced the magnitude of these effects but preserved the retained-base hierarchy, the identity of all three positional anchors, and the overall seed ranking. Combinations of retained positions produced super-additive increases in predicted foldability, although structural-proximity analysis showed that these positions did not consistently form a single compact predicted structural core. Iterative computational selection for lower MFE progressively enriched all three neighborhoods for predicted structure, while six non-conserved biological control 20-mers generated even richer predicted foldability landscapes than the ribosome-inspired seeds. Together, these results show that local RNA sequence space is strongly shaped by starting-sequence identity, nucleotide position, and higher-order retained-position effects and cannot be explained by nucleotide composition or starting-seed structure alone. These conclusions are restricted to MFE-based secondary-structure predictions and do not establish tertiary folding, biochemical function, or experimentally populated structures.

## Introduction

The idea that early life passed through an RNA-dominated stage has focused longstanding attention on ancient catalytic RNA regions, particularly the ribosomal peptidyl transferase center (PTC), which remains an important reference point for models of early RNA-based catalysis and peptide-bond formation (Davidovich et al. 2010; Rivas and Fox 2020; Tirumalai et al. 2021; Bose et al. 2022; Codispoti et al. 2024). Modern ribosomal RNA therefore provides both a functional molecular system and a framework for examining how evolutionarily conserved RNA sequences may constrain access to nearby sequence space.

A central question in RNA evolution is the extent to which a starting sequence influences the secondary structures accessible through mutation. Experimental and computational studies have shown that RNA sequence space is highly non-uniform: different sequences can adopt related folds, random RNA pools can contain compact or structured molecules, and the probability of recovering functional RNAs depends partly on the structural and informational properties of the starting pool (Lee et al. 1993; Joyce 2002; Carothers et al. 2004; Schultes et al. 2005; Tirumalai et al. 2018; Chizzolini et al. 2020). These studies also emphasize that predicted secondary-structure stability is not equivalent to experimentally demonstrated folding or biochemical function. Rather, secondary-structure prediction provides one way to compare the relative thermodynamic accessibility of candidate folds under a common model.

Here, we examined three isolated 20-nucleotide RNA seeds inspired by ribosome-core regions associated with the P-site PTC, a shifted P-loop, and the A-site A-loop. The three sequences were used as conceptual starting points for local sequence-space exploration. They were not intended as exact residue-numbered extracts from a single modern ribosomal RNA reference or as literal ancestral sequences. The analysis therefore asks a limited computational question: under the same mutation scheme and secondary-structure prediction model, do these short seed sequences define different local neighborhoods in their capacity to generate predicted secondary structure?

Because the sequences are only 20 nucleotides long, the present analysis is restricted to simple secondary-structure models. Such short RNAs would not generally be expected to reproduce the complex tertiary architecture of the intact ribosome or other large functional RNAs. We therefore use ViennaRNA minimum-free-energy calculations to estimate secondary-structure accessibility under a standardized thermodynamic model. These calculations do not establish experimentally populated structures, tertiary contacts, ligand binding, catalytic activity, or behavior within the cellular or ribosomal environment.

The three starting sequences also differ in base composition. Set A contains more guanine residues than Sets B and C, whereas the fully mutated libraries generated from each seed have different expected aggregate nucleotide compositions because the native base at every position is excluded from the allowed substitutions. Starting-sequence composition and positional base exclusion are therefore treated as features of the mutational design that may influence predicted foldability, rather than as incidental properties of the sequences.

The study was organized around four related questions. First, do the three ribosome-derived seeds define reproducibly different predicted secondary-structure landscapes? Second, are the observed differences robust across independent random samples and alternative minimum-free-energy thresholds? Third, does iterative computational selection for lower predicted minimum free energy compress the three sequence neighborhoods differently? Fourth, can single- and multiple-position retention analyses identify specific nucleotide positions or retained-position combinations that disproportionately influence predicted foldability?

The iterative selection procedure used here retains the 10% of sequences with the lowest predicted minimum free energies and then introduces further mutations over successive cycles. Because no molecular ligand or binding target is present, this procedure is not experimental SELEX. We therefore refer to it throughout as iterative structure-based computational selection. Experimental SELEX and other iterative-selection studies remain relevant as historical demonstrations that rare RNA functions can be enriched from large sequence pools, but they are not methodologically equivalent to the procedure used here (Tuerk and Gold 1990; Breaker and Joyce 1994).

To distinguish behavior specific to the three ribosome-derived seeds from more general properties of short RNA sequence space, we also analyzed six non-conserved biological control 20-mers. These controls were selected because their unmutated forms lacked stable predicted secondary structure under the same ViennaRNA conditions. The comparison was intended to test whether an initially structured or evolutionarily conserved seed necessarily possesses a richer predicted structural neighborhood than an initially unstructured sequence. It was not intended to establish that one evolutionary class is biologically superior to another.

Together, these analyses provide a controlled comparison of predicted secondary-structure accessibility among short RNA mutational neighborhoods. The conclusions are therefore confined to MFE-based secondary-structure models of isolated 20-nt sequences and to the effects of seed identity, base composition, retained nucleotide position, and computational selection under a shared thermodynamic parameterization.

## Materials and Methods

### Seed Sequences and Design Rationale

**Fig. 1:**
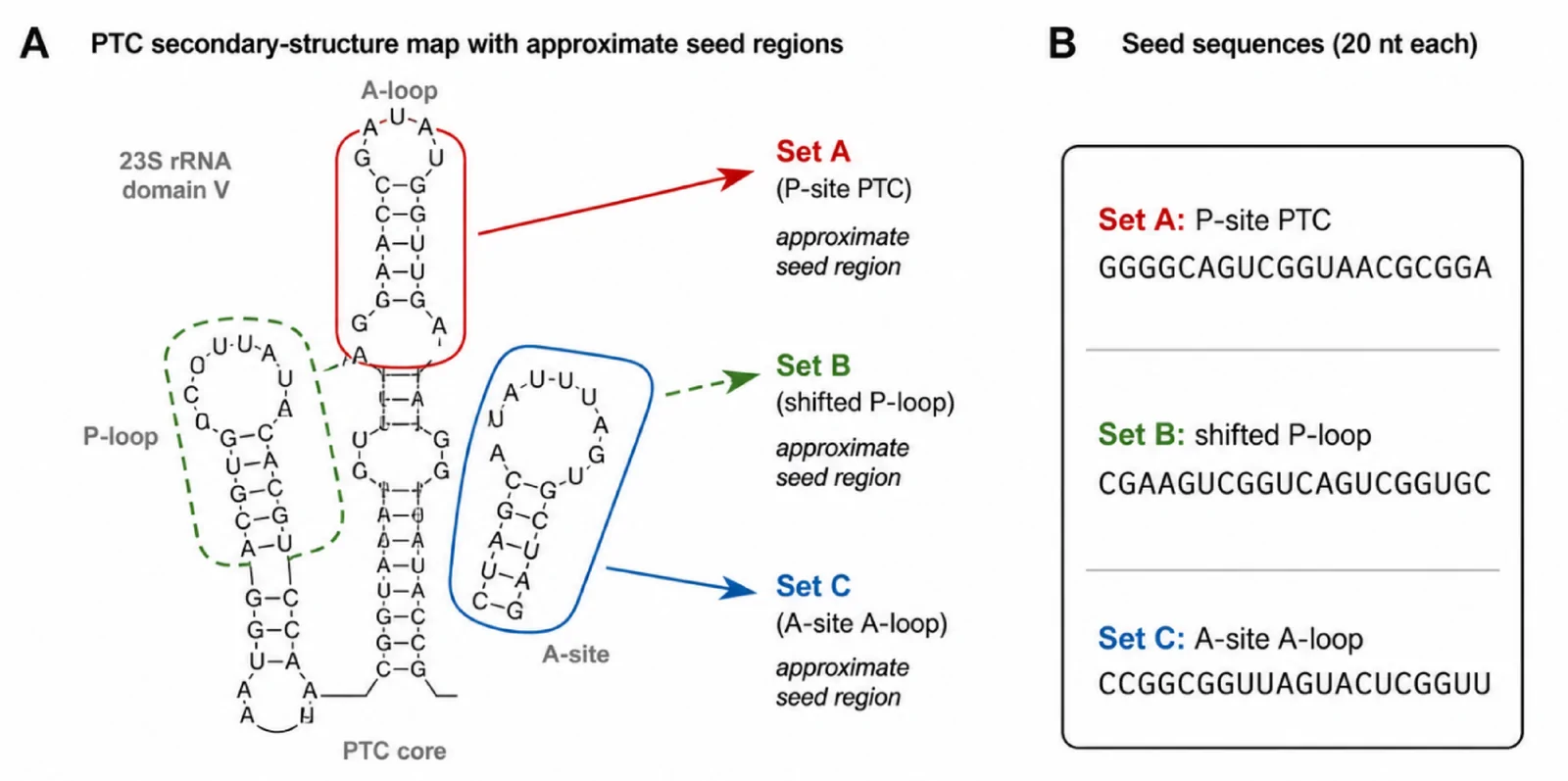
Approximate locations of the ribosome-core-inspired seed regions used in this study relative to the peptidyl transferase center (PTC). **A** Schematic secondary-structure representation of the PTC region, adapted from Tirumalai et al. (2021), showing the approximate locations of the three 20-nt seed regions used in this study: Set A (P-site PTC), Set B (shifted P-loop), and Set C (A-site A-loop). **B** Corresponding seed sequences used for computational sequence-space exploration. The indicated locations are schematic associations and are not intended to represent exact residue-numbered sequence extracts from a single modern rRNA reference

Three 20-nucleotide RNA seed sequences were analyzed: Set A, GGGGCAGUCGGUAACGCGGA; Set B, CGAAGUCGGUCAGUCGGUGC; and Set C, CCGGCGGUUAGUACUCGGUU. These sequences were designed as short ribosome-core-inspired representatives associated conceptually with the P-site peptidyl transferase center, a shifted P-loop region, and the A-site A-loop, respectively. They were not intended as exact residue-numbered excerpts from a single modern ribosomal RNA reference or as reconstructed ancestral sequences. Instead, they served as defined starting points for comparing local mutational neighborhoods under a common computational framework (Davidovich et al. 2010; Rivas and Fox 2020; Tirumalai et al. 2021; Bose et al. 2022). The conceptual relationships of Sets A–C to the corresponding P-site PTC, shifted P-loop, and A-site A-loop regions are illustrated schematically in Fig. 1.

The three seeds differ in nucleotide composition. Set A contains 10 G residues and has 70% GC content, Set B contains eight G residues and has 65% GC content, and Set C contains seven G residues and has 60% GC content. Because each native nucleotide was excluded from the substitutions permitted at its position, the composition of each seed also determined the expected aggregate nucleotide composition of its fully mutated library. Starting-sequence composition and position-specific nucleotide exclusion were therefore treated as features of the experimental design rather than as independent measures of evolutionary age or biological importance.

### Fixed-Length Mutational Libraries

For each 20-nt seed, every nucleotide position was forced to change from its native identity to one of the three non-native nucleotides, selected with equal probability. The resulting theoretical sequence space comprised 3^20^, or approximately 3.49 × 10^9^ possible sequences for each seed. The computational studies sampled subsets of these spaces using the pool sizes described below.

The initial analysis generated 1,000,000 sequences per seed. Subsequent analyses used independent pseudorandom-number-generator seeds to generate non-overlapping replicate pools and larger samples for reproducibility and depth analyses. No sequence in these fully mutated libraries retained the native nucleotide at any of the 20 positions.

For a native base X, the probability of X occurring at that same position in the fully mutated library was zero, whereas each of the other three nucleotides had probability 1/3. Thus, a seed containing more native G residues generated a fully mutated pool in which G was excluded at more positions. For example, although Set A was the most G-rich starting sequence, its fully mutated library had the lowest expected aggregate G frequency among the three sets. Expected nucleotide frequencies were verified against empirical frequencies measured from deposited sequence pools.

The composition analysis supports this point directly: the expected G proportions were 16.7% for Set A, 20.0% for Set B, and 21.7% for Set C, with empirical values closely matching those expectations.

### Secondary-Structure Prediction and Operational Definitions

Secondary structures were predicted using RNAfold version 2.7.2 from the ViennaRNA package (Hofacker 2003; Lorenz et al. 2011; Varenyk et al. 2023). Calculations used the bundled Turner 2004 nearest-neighbor parameter set at 37 °C (Serra and Turner 1995; Tinoco and Bustamante 1999). No custom energy-parameter file was supplied. The default ViennaRNA monovalent-salt setting of 1.021 M was used because no explicit --salt option was specified. Default dangling-end treatment (d2) and allowance of isolated base pairs were retained.

The standard RNAfold minimum-free-energy algorithm predicts pseudoknot-free secondary structures. G-quadruplex prediction was not enabled, and therefore G-quadruplex structures were outside the modeled structure space. The calculations were performed on isolated 20-nt sequences and did not include tertiary interactions, ligand binding, molecular crowding, proteins, intact ribosomal context, or cellular ionic conditions.

The following operational definitions were used:

- Predicted secondary structure: the minimum-free-energy dot-bracket structure returned by RNAfold.
- Minimum free energy: the calculated free energy of the RNAfold-predicted secondary-structure model under the stated parameterization.
- Predicted stable sequence: a sequence with MFE below the specified threshold.
- Predicted foldability: the fraction of sequences in a pool with MFE below that threshold.
- Primary stability threshold: MFE < − 2 kcal/mol.
- Positional anchor: the retained nucleotide position producing the highest predicted foldability within a seed.
- Cooperative retained-position set: a combination of retained positions whose observed predicted foldability exceeded the specified linear-additive expectation.

Because these are computational thermodynamic estimates, “predicted stable” and “predicted foldability” refer only to secondary-structure behavior under the ViennaRNA model. They do not demonstrate experimentally populated conformations, tertiary structures, biochemical activity, or biological function. The exact settings recovered from the pipeline are RNAfold 2.7.2, Turner 2004 parameters, 37 °C, default 1.021 M monovalent salt, default dangling-end treatment, G-quadruplexes not modeled, and pseudoknots not modeled.

### Threshold-Sensitivity Analysis

The primary predicted-foldability measure used an MFE threshold of − 2 kcal/mol. To test whether the comparative results depended on this permissive threshold, predicted foldability was recalculated at − 3, −4, and − 5 kcal/mol using the deposited per-sequence MFE values. Rank order among the three ribosome-derived seeds and comparisons with the biological controls were evaluated across all four thresholds.

### Iterative Structure-Based Computational Selection

Each seed background began with 100,000 sequences. In each of eight computational-selection cycles, sequences were ranked by predicted MFE, and the 10% with the lowest MFE values were retained. The retained sequences were then replicated and subjected to a 5% mutation rate to restore the population size for the next cycle. The objective function was therefore lower predicted MFE rather than ligand-binding affinity or biochemical function. Additional sensitivity runs were performed at mutation rates of 1%, 2.5%, and 10%. These runs used initial populations of 30,000 sequences to reduce computational cost. Because pool size differed from the original 5% analysis, absolute MFE values were not compared directly among mutation-rate conditions; the sensitivity analysis was used to compare seed ranking and qualitative convergence behavior. The procedure is described here as iterative structure-based computational selection. Experimental SELEX and other iterative-enrichment experiments are relevant background for understanding sequence-space selection, but the present procedure is not methodologically equivalent because it lacks ligand-directed selection (Tuerk and Gold 1990; Breaker and Joyce 1994).

### Positional-Retention Analysis

For each seed, 20 single-position-retention sub libraries were generated. In each sub library, one native nucleotide was retained while the remaining 19 positions were forced to mutate to one of their three non-native alternatives. Each sub library contained 50,000 sequences, giving 60 sub libraries in total. Predicted secondary structures and MFE values were calculated under the same ViennaRNA settings used for the fully mutated pools. Predicted foldability was defined as the fraction of sequences with MFE < − 2 kcal/mol, with additional threshold analyses as described above. The strongest position within each seed was designated its positional anchor. Because all three strongest anchors retained G, each anchor was also compared with the other G-retained positions within the same seed. This same-base analysis was used to distinguish a position-specific effect from a general retained-guanine effect.

### G·U-Wobble Sensitivity Analysis

To test whether the retained-guanine effect depended on ViennaRNA’s treatment of G·U wobble pairing, the deposited sequence libraries were refolded using RNAfold 2.7.2 with the native --noGU option. This option excludes G·U pairs from the folding recursion. All other settings were held constant, including parameter set, temperature, default salt concentration, dangling-end treatment, and allowance of isolated base pairs.

No new sequences were generated. The comparison used:

- All 60 single-position-retention sub libraries;
- Six 1,000,000-sequence fully mutated replicate pools for each seed;
- The best-performing five-position retained set for each seed.

For the single-position libraries, the default and --noGU models were compared for:

- Mean predicted foldability by retained base;
- Identity of the strongest position in each seed;
- Anchor-versus-other-G differences;
- Per-position change in predicted foldability.

For the baseline pools, the analysis tested whether the Set B > Set C > Set A ranking was preserved. For the five-position sets, super-additivity was recalculated using all baseline and retained-position inputs folded under --noGU.

### Higher-Order Retained-Position Analysis

Combinations of three, four, and five retained positions were evaluated to test whether predicted foldability increased beyond the contribution observed for individual retained positions. The best-performing combination at each value of *k* was identified within each seed. A linear-additive expectation was calculated from the fully mutated baseline and the marginal contribution of each corresponding single retained position. An observed multi-position predicted-foldability value greater than this expectation was classified operationally as super-additive. The term cooperative retained-position set refers to this statistical super-additivity. It does not, by itself, imply that all retained nucleotides directly contact one another or form a single compact physical structure.

### Structural-Proximity Analysis of Five-Position Retained Sets

To evaluate whether retained positions in the best five-position sets were proximal in the predicted secondary structures, the previously calculated MFE dot-bracket structures from all 2,000 sequences in each five-position pool were parsed.

For every pair of retained positions, the relationship was classified as:

- Directly paired: the two retained positions formed a predicted base pair with each other;
- Same stem: both were paired within the same helix but not directly to each other;
- Adjacent: the positions occurred in immediately connected structural elements or within a shared enclosing loop;
- Separated: no relationship closer than the above categories was present.

The analysis was performed across all 2,000 predicted MFE structures in each pool rather than from a single representative structure.

### Biological Controls

Six non-conserved biological control 20-mers were analyzed under the same fixed-length mutation and computational-selection procedures. These comprised one sequence derived from the *Escherichia coli* MicF small RNA (Mizuno et al. 1984) and five sequences derived from bacterial and eukaryotic rRNA hypervariable regions. The identities and source regions of all controls are listed in Table 1. The control sequences were selected because their unmutated 20-nt forms lacked stable predicted secondary structure under the same RNAfold conditions. Their inclusion tested whether an initially structured or evolutionarily conserved seed necessarily defines a mutational neighborhood with greater predicted secondary-structure accessibility. The control comparison was not intended to establish evolutionary superiority, ancestral status, or biological function. It compared only predicted secondary-structure properties of short mutational neighborhoods generated under the same computational rules.

**Table 1:** Sets A–C were designed as conceptual ribosome-core-inspired 20-nt starting sequences and were not intended as exact residue-numbered extracts from a single modern rRNA reference

Group	Name	Conceptual source/region	20-nt sequence
Ribosome-core-inspired seed	Set A	P-site peptidyl transferase center-inspired	GGGGCAGUCGGUAACGCGGA
Ribosome-core-inspired seed	Set B	Shifted P-loop-inspired	CGAAGUCGGUCAGUCGGUGC
Ribosome-core-inspired seed	Set C	A-site A-loop-inspired	CCGGCGGUUAGUACUCGGUU
Biological control	MicF	*E. coli* MicF small RNA	UUUCAACCGGAUGCCUCGCA
Biological control	ec_16S_V4	*E. coli* 16 S rRNA V4 region	ACUGCUAGGAUCACGACGUU
Biological control	bs_16S_V4	*Bacillus subtilis* 16 S rRNA V4 region	AUGAACUACGACGACGACUA
Biological control	tm_16S_V4	*Thermotoga maritima* 16 S rRNA V4 region	ACGAUGACGACUACGACGAC
Biological control	sc_18S_V9	*Saccharomyces cerevisiae* 18 S rRNA V9 region	ACGAACGACGACAACGACAA
Biological control	hs_18S_V9	*Homo sapiens* 18 S rRNA V9 region	AUGAACGACUACGACAACGA

The biological controls comprised one MicF-derived sequence and five sequences derived from rRNA hypervariable regions

### Infernal/Rfam Screening

Subsets of the fixed-length mutational libraries were screened for homology to known RNA families using Infernal (Nawrocki and Eddy 2013) against the Rfam database (Ontiveros-Palacios et al. 2025). The same screening framework was applied to the ribosome-core-inspired seeds and biological controls. Because Rfam matches were rare, these searches were used as an exploratory comparison of RNA-family similarity and were not interpreted as evidence of biochemical function.

### Statistical Analysis

Replicate-pool differences among seed sets were evaluated using Welch one-way ANOVA, followed by pairwise Welch t-tests with Holm–Bonferroni correction (Welch 1951; Holm 1979). Effect sizes were reported as Cohen’s d and Cliff’s δ (Cohen 1988; Cliff 1993). Reproducibility across independent pools was summarized as mean ± SD, coefficient of variation (CV), and 95% confidence intervals on the mean.

For the G·U-wobble sensitivity analysis, differences between each anchor and the other G-retained positions within the same seed were evaluated using two-proportion z-tests. Extremely small, calculated p-values that fell below double-precision numerical reporting limits were reported as *p* < 10^− 300^, rather than as zero. For single-position retention data, retained-base classes (G/C/U/A) were compared using Kruskal–Wallis tests, followed by pairwise Mann–Whitney U tests with Holm correction (Mann and Whitney 1947; Kruskal and Wallis 1952; Holm 1979). Within-seed comparisons of each anchor with the pooled set of other G-retained positions were evaluated using two-proportion z-tests and Wilson 95% confidence intervals (*n* = 50,000 per sub library) (Wilson 1927).

For multi-position retention, monotonic increases in predicted foldability across k = 3, 4, and 5 were tested using the Jonckheere–Terpstra trend test (Terpstra 1952; Jonckheere 1954). Cooperativity of the best k = 5 combinations was evaluated relative to a linear-additive null model. If $\:{p}_{0}$ denotes the predicted foldability of the fully mutated background and $\:{p}_{i}$the predicted foldability observed when only retained position $\:i\:$is fixed, then the additive expectation for a retained set * S* was defined as.

$$p_{{add}} (S)\, = \,p_{0} \, + \,\sum\limits_{{i \in \,S}} {\left( {p_{i} \, - \,p_{0} } \right)}$$

Observed multi-position predicted foldability values exceeding $\:{p}_{\mathrm{add}}\left(S\right)\:$were interpreted as evidence of super-additive cooperativity among retained positions. Deviations from this expectation were assessed using one-sample z-tests. Statistical calculations were performed in Python using SciPy, NumPy, and pandas (McKinney 2010; Virtanen et al. 2020; Harris et al. 2020). A summary of the computational analyses performed in this study is provided in Table 2.   

**Table 2:** Summary of computational analyses performed in this study

Analysis	Pool design	Primary outcome	Purpose
Baseline mutational libraries	1,000,000 sequences per seed	Predicted foldability and MFE distribution	Compare local neighborhoods
Reproducibility	Independent replicate pools	Mean, SD and rank order	Test stochastic robustness
Threshold sensitivity	MFE < − 2 to − 5 kcal/mol	Rank-order preservation	Test cutoff dependence
Computational selection	Eight iterative cycles	MFE trajectory and predicted-stable fraction	Test response to MFE selection
Single-position retention	20 × 50,000 sequences per seed	Position-specific foldability	Identify anchors
G·U sensitivity	Existing pools refolded with --noGU	Ranking and anchor preservation	Test model dependence
Higher-order retention	(k = 3, 4, 5) retained positions	Super-additivity	Test retained-position cooperation
Structural proximity	2,000 structures per best (k = 5) pool	Pair/stem/adjacency classification	Assess predicted structural proximity
Biological controls	Six control 20-mers	Baseline and computational-selection metrics	Compare ribosome-core-inspired and control neighborhoods

## Results

Fixed-length 20-nt libraries reveal reproducible seed-dependent differences in predicted secondary-structure accessibility. The three ribosome-derived seeds produced distinct local distributions of ViennaRNA-predicted minimum free energies under the same full-mutation scheme. In the initial 1,000,000-sequence libraries, Set B showed the highest predicted foldability, defined as the fraction of sequences with MFE < − 2 kcal/mol. The predicted-stable fractions were 13.5% for Set A, 18.3% for Set B, and 17.0% for Set C.

Across independent replicate pools, the same ranking was preserved. The coefficients of variation for predicted foldability were 1.29% for Set A, 0.89% for Set B, and 0.45% for Set C. The order Set B > Set C > Set A therefore remained reproducible across independently generated pools and was not attributable to a single pseudorandom sample.

Increasing sampling depth to 10 million sequences per seed did not materially alter the central distributions. Predicted foldability remained approximately 13.4% for Set A, 18.5% for Set B, and 16.9% for Set C. Deeper sampling primarily extended the low-MFE tails rather than changing the comparative ranking among seeds.

**Fig. 2:**
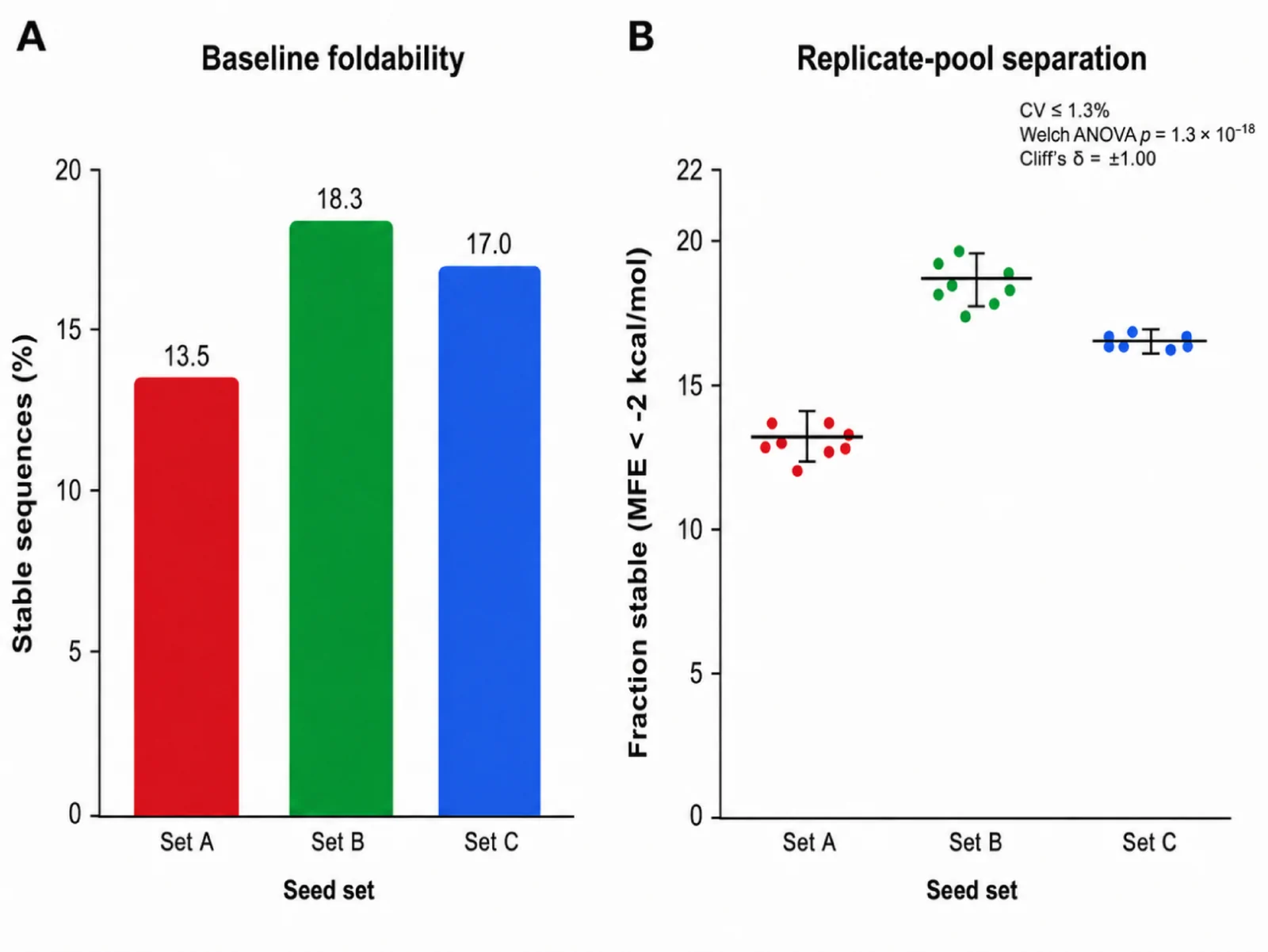
Baseline predicted foldability and reproducibility across independent replicate pools. **A** Baseline predicted foldability for the three ribosome-core-inspired seed sets, expressed as the percentage of sequences with RNAfold-predicted minimum free energy (MFE) < − 2 kcal/mol. Set B showed the highest predicted foldability (18.3%), followed by Set C (17.0%) and Set A (13.5%). **B** Predicted-stable fractions across independent replicate pools. Points represent individual replicate pools, with horizontal bars indicating the mean and error bars indicating variability among replicates. The Set B > Set C > Set A ranking was preserved across replicates, with low coefficients of variation (CV ≤ 1.3%) and a significant overall difference among seed sets by Welch one-way ANOVA (*p* = 1.3 × 10⁻¹⁸)

These results concern predicted secondary-structure accessibility within isolated 20-nt mutational neighborhoods. They do not establish experimentally populated structures or the folding behavior of these sequences within intact ribosomal RNA. The fixed-length comparisons and their reproducibility across replicate pools are summarized in Fig. 2.

### Single-Position Retention Identifies Reproducible Positional Anchors

To determine whether seed-dependent differences were distributed across the full sequence or concentrated at particular positions, 20 single-position-retention sub libraries were analyzed for each seed.

Across the 60 sub libraries, the mean predicted-foldability hierarchy by retained nucleotide was: *G > C > U > A*. Retained-G sub libraries had the highest mean predicted foldability, whereas retained-A sub libraries had the lowest. The strongest retained position in each seed was:

- Set A: position 16, G.
- Set B: position 5, G.
- Set C: position 17, G.

**Fig. 3:**
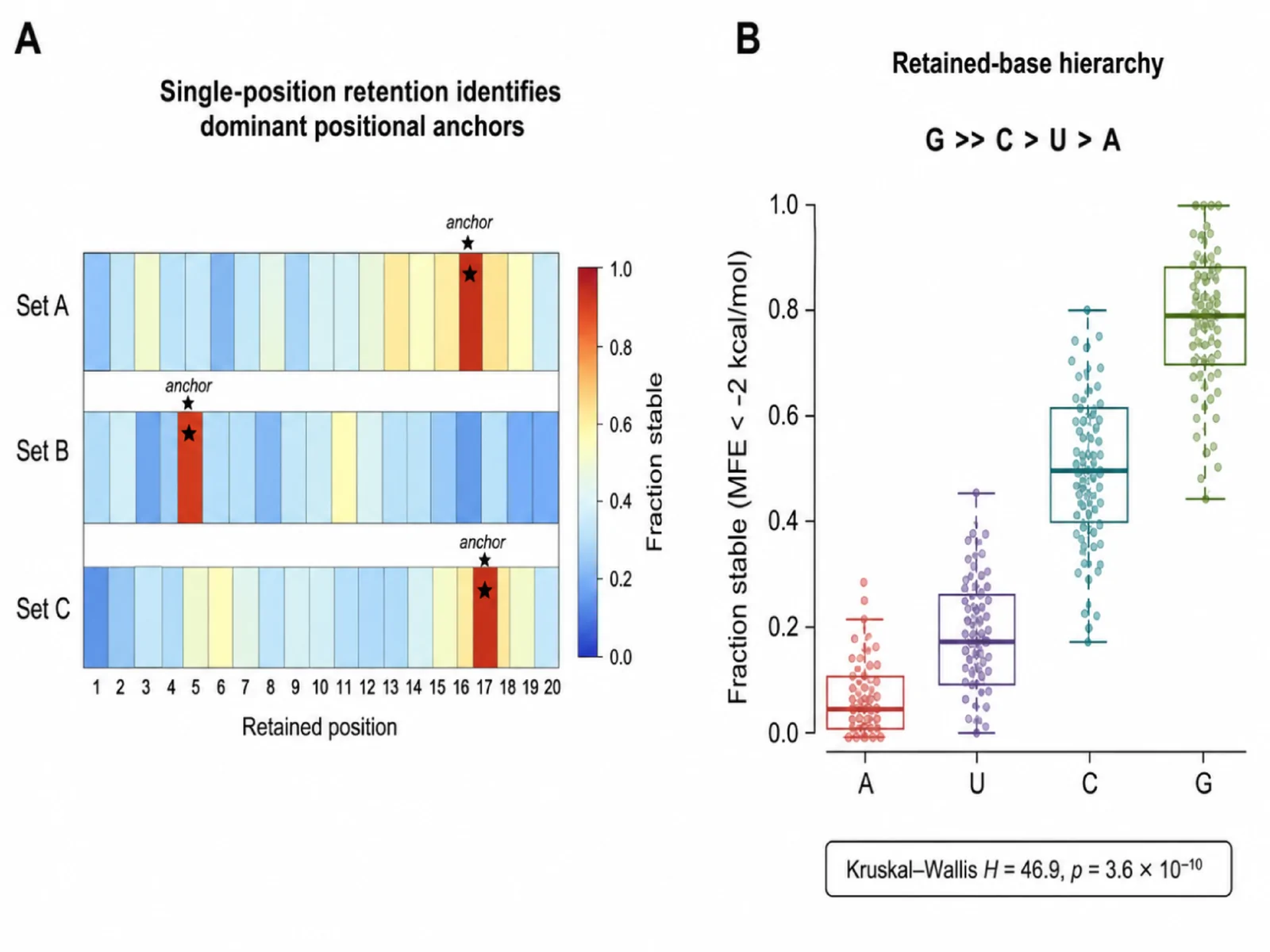
Single-position retention identifies positional anchors and a retained-base hierarchy in predicted foldability. **A** Heat map showing predicted foldability for each single-position-retention sublibrary across Sets A–C, expressed as the fraction of sequences with RNAfold-predicted MFE < − 2 kcal/mol. The strongest position within each seed is marked as the positional anchor: position 16 in Set A, position 5 in Set B, and position 17 in Set C. **B** Distribution of predicted foldability values grouped by the identity of the retained nucleotide across all single-position-retention sublibraries. Retained-base classes followed the hierarchy G > C > U > A, with a significant overall difference among groups by Kruskal–Wallis test (H = 46.9, *p* = 3.6 × 10⁻¹⁰)

Because the strongest position in every seed retained guanine, each was compared directly with the other G-retained positions within the same seed. Each anchor remained significantly higher than the pooled set of other G-retained positions within the same seed: Set A position 16 by + 5.91% points (*p* < 10^− 300^), Set B position 5 by + 3.37% points (*p* < 10^− 300^), and Set C position 17 by + 3.28% points (*p* < 10^− 300^). Thus, the positional-anchor pattern was not reducible to guanine identity alone but reflected a combination of retained base and seed-specific positional context. The retained-base hierarchy and position-specific foldability values are summarized in Fig. 3.

### Non-Conserved Biological Controls Define More Foldable Predicted Secondary-Structure Neighborhoods

The control panel comprised the *Escherichia coli* MicF-derived 20-mer (Mizuno et al. 1984) and five 20-mers derived from bacterial and eukaryotic rRNA hypervariable regions. Their unmutated forms lacked stable predicted secondary structure under the same RNAfold conditions used for Sets A-C.

Despite beginning from unstructured predicted seed states, all six controls produced more foldable mutational neighborhoods than the three ribosome-derived seeds. Baseline predicted foldability ranged from 25.23% for the *E. coli* 16 S V4 control to 32.89% for the human 18 S V9 control, compared with 18.27% for Set B, the highest of the three ribosome-derived seeds.

The control results therefore show that predicted structure in the unmutated starting sequence is not sufficient to predict the overall predicted secondary-structure richness of its fully mutated neighborhood. This comparison should not be interpreted as showing that the controls are biologically more important or evolutionarily superior. Rather, it demonstrates that seed-state predicted structure and neighborhood-level predicted foldability are separable properties.

### Multiple Retained Positions Produce Super-Additive Effects on Predicted Foldability

**Fig. 4:**
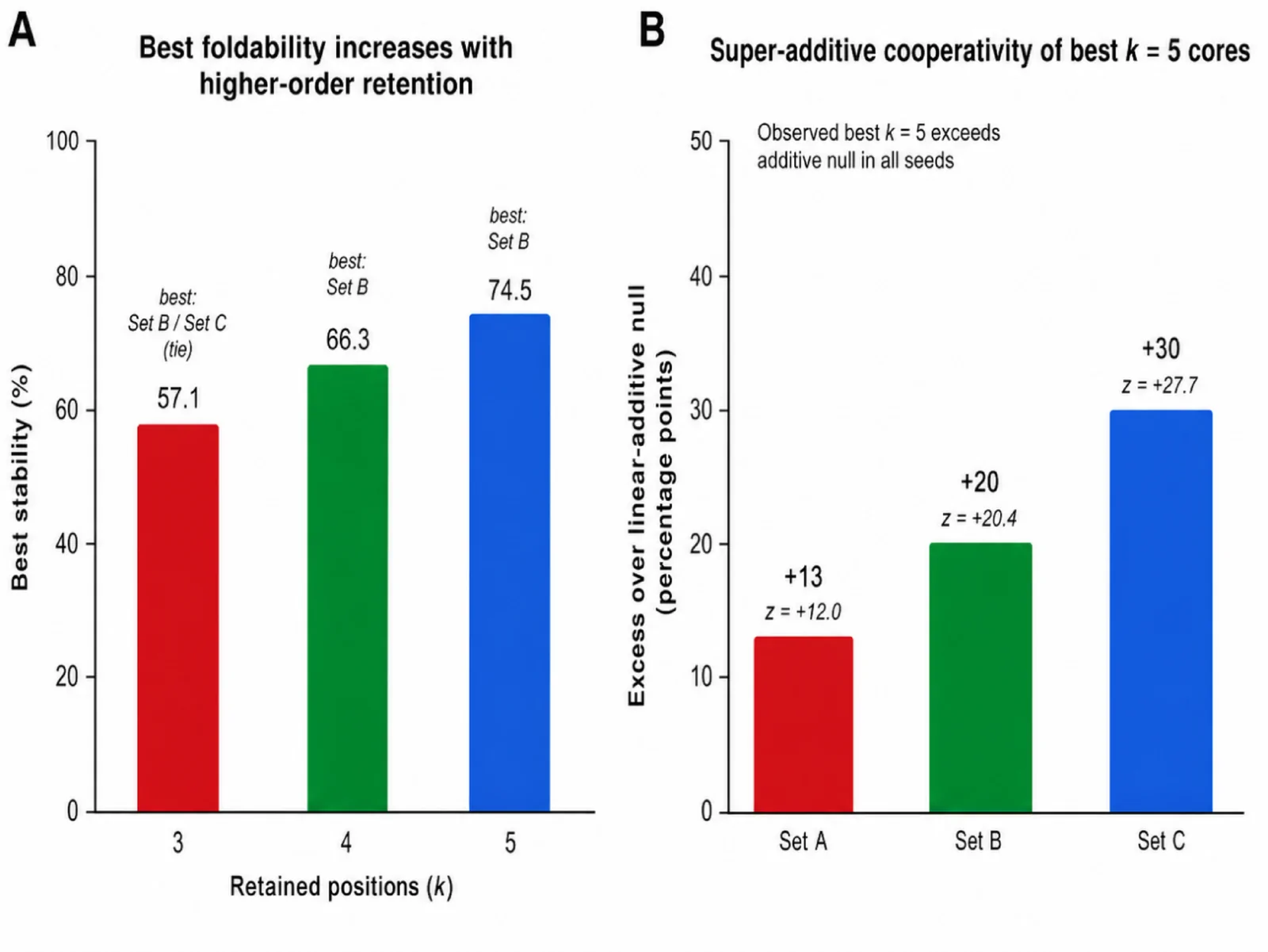
Higher-order positional retention increases predicted foldability and produces super-additive effects. **A** Best predicted foldability observed among three-, four-, and five-position retention combinations, expressed as the percentage of sequences with RNAfold-predicted MFE < − 2 kcal/mol. Maximum foldability increased with the number of retained positions, reaching 57.1% for the best combination, 66.3% for the best combination, and 74.5% for the best combination. **B** Excess predicted foldability of the best five-position retained set in each seed relative to the linear-additive expectation calculated from the fully mutated baseline and corresponding single-position effects. All three seeds exceeded the additive null, indicating super-additive cooperativity among retained positions

The single-position analysis was extended to combinations of three, four, and five retained positions. Predicted foldability increased with the number of retained positions in all three seeds. The best-performing five-position set was found in Set B and reached approximately 74.5% predicted foldability under the default RNAfold model. To test whether these increases could be explained by adding the individual single-position effects, observed five-position results were compared with a linear-additive expectation. The best five-position set in each seed exceeded that expectation, indicating statistical super-additivity (Fig. 4). 

 Under the --noGU model, the best five-position sets remained super-additive:

- Set A: observed 44.25%, additive prediction 32.38%.
- Set B: observed 55.85%, additive prediction 34.21%.
- Set C: observed 60.45%, additive prediction 27.73%.

The corresponding z values ranged from + 11.34 to + 32.68, with all comparisons *p* < 10^− 300^. Thus, the best five-position retained sets remained super-additive even when G·U wobble pairing was excluded .

### Iterative Structure-Based Computational Selection Enriches Low-MFE Sequences

Because no ligand or molecular binding target was used, this procedure is referred to here as iterative structure-based computational selection rather than SELEX. Under the original 5% mutation condition, all three ribosome-core-inspired seed populations became strongly enriched for sequences meeting the MFE-based predicted-stability criterion over eight computational-selection cycles. By the final cycle, each population exceeded 99.5% predicted-stable sequences.

**Fig. 5:**
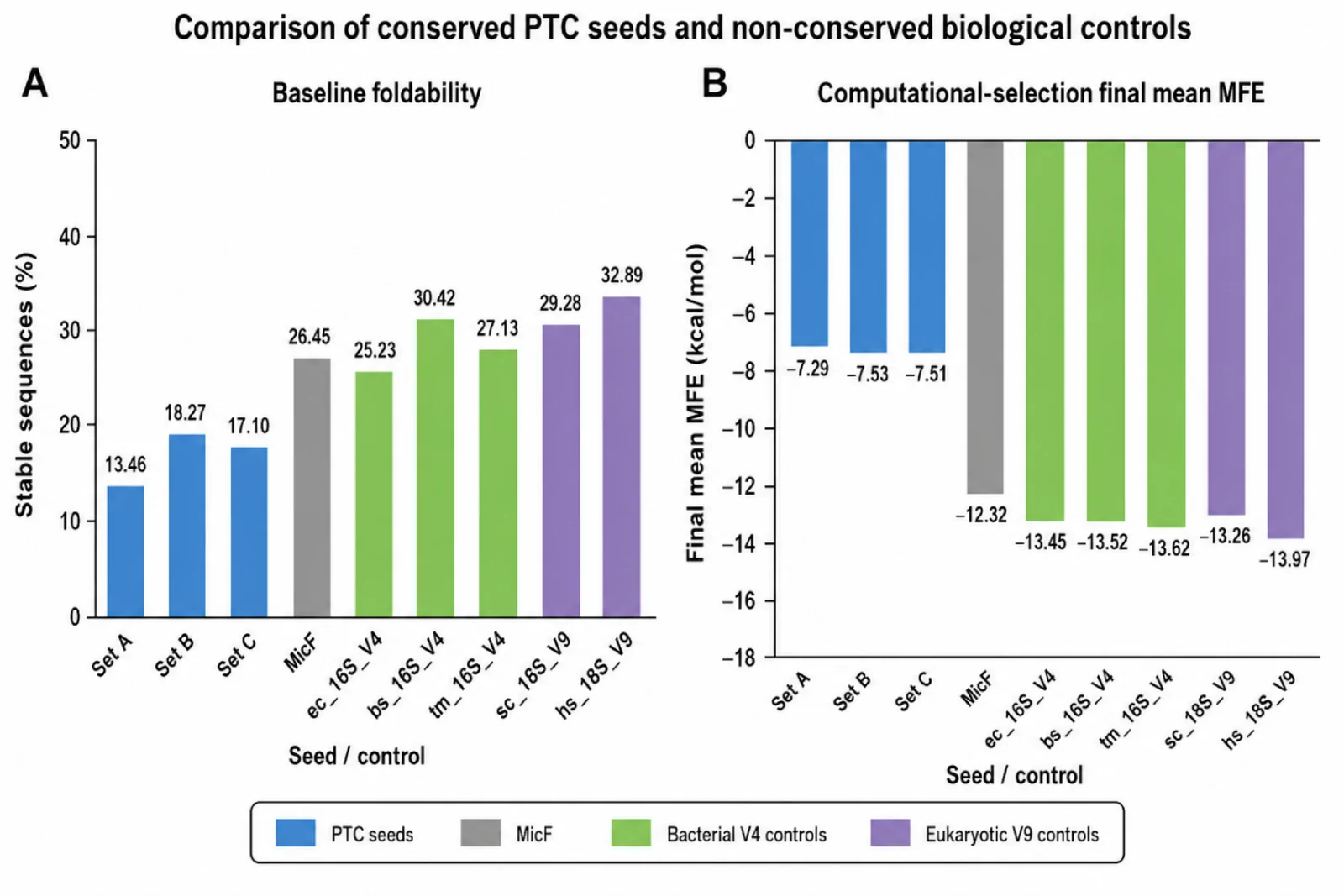
Comparison of ribosome-core-inspired seeds with non-conserved biological controls. **A** Baseline predicted foldability, expressed as the percentage of sequences with RNAfold-predicted MFE < − 2 kcal/mol, for Sets A–C and six biological control 20-mers. All six controls showed greater baseline predicted foldability than the three ribosome-core-inspired seeds. **B** Final mean MFE after iterative structure-based computational selection for the same seed and control sets. The biological controls reached substantially more negative final mean MFE values than Sets A–C under the same computational-selection framework

Set B reached the most negative mean MFE under the original 5% condition, whereas Set A showed the largest absolute increase relative to its starting population. These results indicate that repeated retention and mutation under an MFE-based objective strongly compress the predicted secondary-structure distributions of all three seed backgrounds. The comparison of baseline predicted foldability and final mean MFE between the ribosome-core-inspired seeds and biological controls is summarized in Fig. 5.

The additional mutation-rate analyses showed that this qualitative convergence was robust across rates of 1%, 2.5%, 5%, and 10%, with all populations reaching at least 97% predicted-stable sequences. Set A remained the least favorable background at every tested rate. The relative ordering of Sets B and C varied with mutation rate, indicating that the finer ranking between those two seeds depends on the computational-selection regime. Because the sensitivity runs used a smaller initial population than the original 5% analysis, absolute MFE values should not be compared across mutation-rate conditions. The relevant comparison is the within-condition ranking among seed backgrounds .

### The Seed Ranking is Preserved Across Stricter MFE Thresholds

**Fig. 6:**
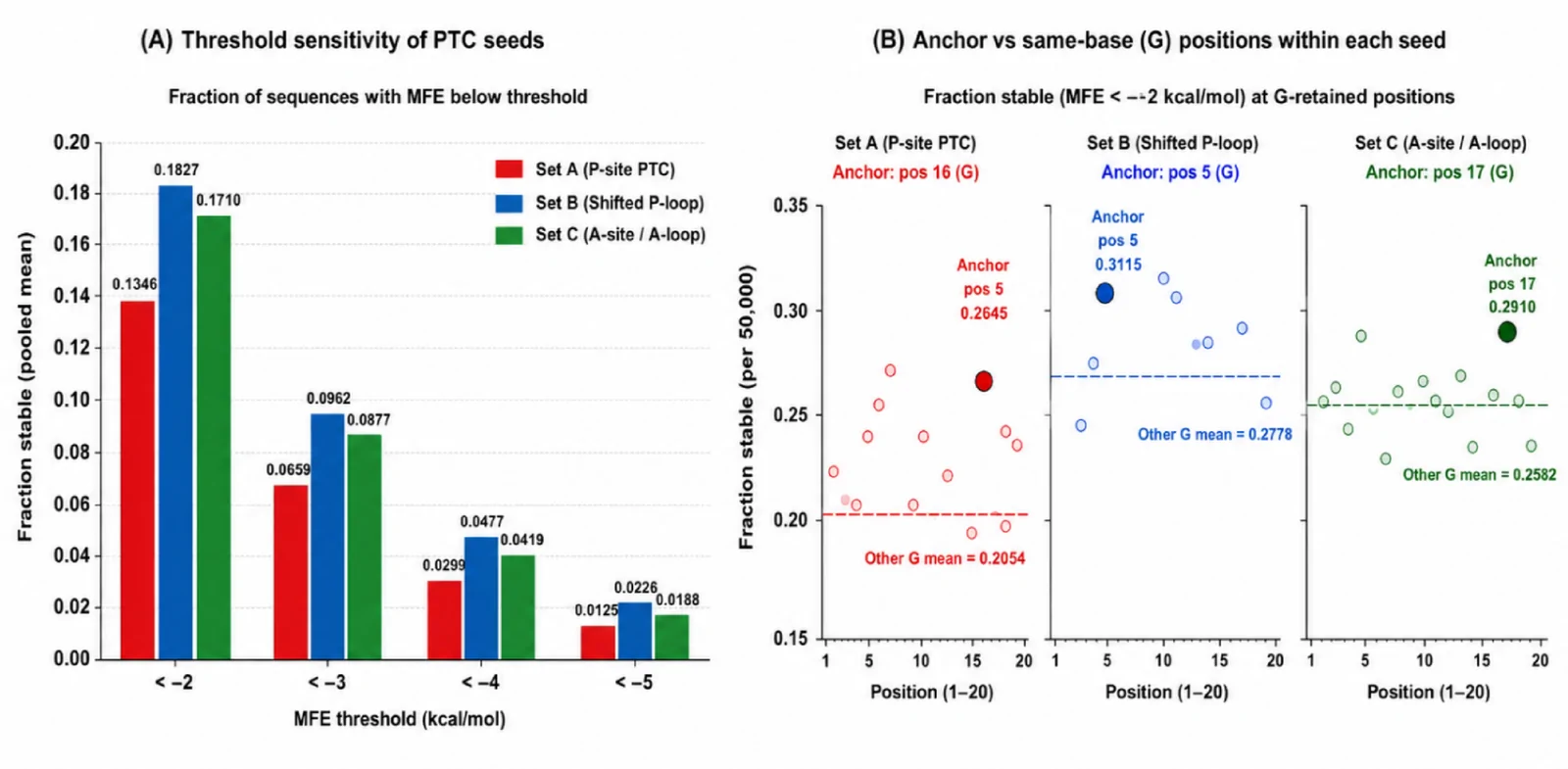
Threshold robustness and anchor specificity in the ribosome-core-inspired seeds. **A** Threshold sensitivity of predicted foldability for Sets A-C. The fraction of sequences with RNAfold-predicted MFE below − 2, −3, −4, and − 5 kcal/mol is shown for each seed. The ranking Set B > Set C > Set A was preserved across all four thresholds. **B** Comparison of the strongest retained-G positional anchor with the other G-retained positions within each seed at the primary MFE < −2 kcal/mol threshold. The identified anchors were position 16 in Set A, position 5 in Set B, and position 17 in Set C. In each seed, the anchor showed higher predicted foldability than the pooled set of other G-retained positions, supporting a position-specific effect beyond retained-guanine identity alone

To test whether the results depended on the primary MFE < − 2 kcal/mol criterion, predicted foldability was recalculated at thresholds of − 3, −4, and − 5 kcal/mol. The rank order Set B > Set C > Set A was preserved at every threshold. At MFE < − 2 kcal/mol, the predicted-stable fractions were 0.1346, 0.1827, and 0.1710 for Sets A, B, and C, respectively. At MFE < − 3 kcal/mol, the corresponding fractions were 0.0659, 0.0962, and 0.0877; at MFE < − 4 kcal/mol, 0.0299, 0.0477, and 0.0419; and at MFE < − 5 kcal/mol, 0.0125, 0.0226, and 0.0188. Thus, the comparative seed ranking was not an artifact of the permissive primary threshold, although the absolute fraction of sequences meeting the predicted-stability criterion declined as the threshold became more stringent. Threshold-dependent foldability for Sets A–C is summarized in Fig. 6A, with corresponding values for the biological controls provided in Table S1. The same-base comparison of each positional anchor with the other G-retained positions within the corresponding seed is shown in Fig. 6B.

### G·U Wobble Pairing Contributes to the Magnitude, But Not the Positional Specificity, of the Retained-G Effect

To determine whether the retained-G effect depended on ViennaRNA’s treatment of G·U wobble pairing, all deposited baseline, single-position-retention, and best five-position libraries were refolded with G·U pairing disabled using RNAfold --noGU.

Removing G·U wobble pairs reduced predicted foldability across all retained-base classes. Mean predicted foldability declined from 24.84% to 16.56% for retained G, from 16.11% to 11.94% for retained C, from 14.39% to 9.40% for retained U, and from 11.59% to 8.25% for retained A. Nevertheless, the hierarchy G > C > U > A remained unchanged.

The strongest position in each seed was also unchanged:

- Set A position 16: 26.45% default, 15.95% --noGU.
- Set B position 5: 31.15% default, 20.94% --noGU.
- Set C position 17: 29.10% default, 19.99% --noGU.

Each anchor remained significantly higher than the other G-retained positions within the same seed under --noGU: +2.83% points for Set A, + 1.85 for Set B, and + 2.41 for Set C, with all comparisons *p* < 10^− 300^. The fully mutated baseline pools showed the same robustness. Disabling G·U pairing reduced predicted foldability from 13.46% to 8.87% for Set A, from 18.27% to 12.80% for Set B, and from 17.10% to 11.91% for Set C. The original Set B > Set C > Set A order was therefore preserved.

**Fig. 7:**
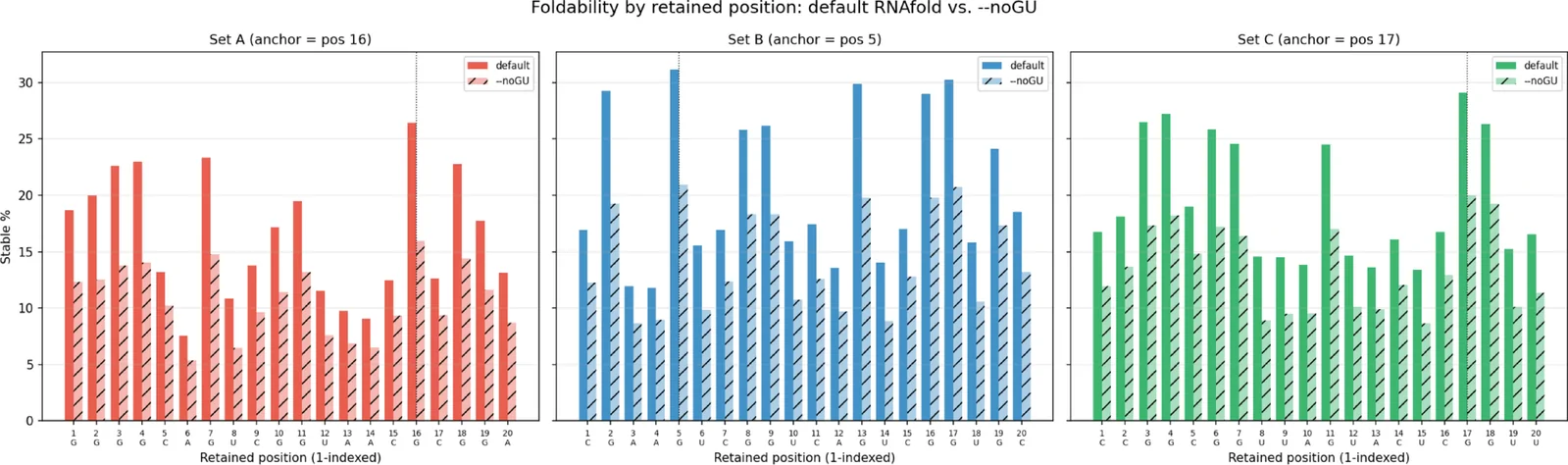
Effect of disabling G·U wobble pairing on position-specific predicted foldability. Predicted foldability for each single-position-retention sublibrary is shown under the default RNAfold model and with G·U wobble pairing disabled using --noGU for Set A, Set B, and Set C. Bars represent the percentage of sequences with RNAfold-predicted MFE < − 2 kcal/mol at each retained position. Disabling G·U pairing reduced predicted foldability across positions but preserved the identity of the strongest positional anchor in each seed: position 16 in Set A, position 5 in Set B, and position 17 in Set C

These results show that G·U wobble pairing contributes substantially to the absolute magnitude of predicted foldability, particularly for retained-G sub libraries, but does not account for the overall seed ranking or the position-specific identity of the three anchors. Per-position predicted foldability under default RNAfold and --noGU conditions is shown in Fig. 7.

### Retained Positions Show Partial, But Not Universal, Proximity in Predicted Secondary-Structure Models

To address whether the positions within the best five-position sets were structurally proximal in predicted secondary structure, all pairwise relationships were classified across the 2,000-sequence pool for each seed. Some retained-position pairs frequently occupied the same predicted helix or paired directly. For example:

- Set A positions 3 and 4 occurred in the same stem in 52.0% of sequences.
- Set B positions 2 and 15 paired directly in 31.0% of sequences.
- Set C positions 3 and 4 occurred in the same stem in 67.8% of sequences.

However, other retained-position pairs were separated in a substantial fraction of predicted structures. Thus, the five retained positions did not consistently form one compact structural unit across the full pools.

The appropriate conclusion is that the retained-position sets are statistically cooperative in their effects on predicted foldability, while their structural proximity is partial and ensemble-dependent. The most frequent direct-pair and same-stem relationships are summarized in Table 3.

**Table 3:** Representative predicted structural relationships among retained positions in the best five-position sets

Seed	Retained-position relationship	Frequency
Set A	Positions 3 and 4 in same stem	52.0%
Set B	Positions 2 and 15 directly paired	31.0%
Set C	Positions 3 and 4 in same stem	67.8%

## Discussion

### Short Ribosome-Derived Seeds Define Non-Equivalent Predicted Secondary-Structure Neighborhoods

The principal result of this computational study is that the three ribosome-core-inspired 20-nt seeds define reproducibly different local distributions of ViennaRNA-predicted secondary-structure stability. Across independent fixed-length mutational libraries, Set B consistently produced the highest fraction of sequences meeting the prespecified MFE-based stability criterion, followed by Set C and Set A. This ranking persisted across replicate pools, deeper sampling, and progressively stricter MFE thresholds.

These findings should be interpreted as differences in predicted secondary-structure accessibility under a shared thermodynamic model, not as evidence that the isolated 20-nt sequences adopt the predicted structures experimentally or that the corresponding regions behave similarly within intact ribosomal RNA. The short sequence length permits comparison of simple stem-loop and duplex-like secondary-structure models, but it does not support analysis of the complex tertiary architecture, long-range interactions, protein contacts, or ionic environment of the complete ribosome.

The present results therefore address a limited question: whether different short starting sequences generate mutational neighborhoods with different distributions of predicted MFE values under identical computational conditions. Within that scope, the strong reproducibility of the ranking indicates that seed identity influences the model-predicted local landscape.

### Relationship to Previous Studies of Random RNA Pools and Sequence–Structure Accessibility

The results are consistent with earlier experimental and computational work showing that RNA sequence space is heterogeneous and that the structural and functional properties recoverable from a random or partially constrained pool depend on the organization of that pool. Experimental studies have shown that random RNA sequences can contain molecules with reproducible secondary structure and compact folding behavior, and that enrichment for folded or compact RNAs can increase the probability of recovering biochemical function (Carothers et al. 2004; Schultes et al. 2005; Chizzolini et al. 2020).

Those studies differ fundamentally from the present analysis because they evaluated experimentally folded or functional molecules, whereas the current study compares ViennaRNA-predicted MFE secondary structures. Nevertheless, they provide relevant context for the broader principle that RNA sequence space is not uniform and that the accessibility of structured or functional molecules depends partly on the sequence pool from which selection begins.

The present results extend that general sequence-space concept at the level of short, fully mutated neighborhoods. They show that even when sequence length, mutation rules, computational model, and sampling depth are held constant, different 20-nt starting sequences can generate distinct predicted secondary-structure distributions.

### Iterative Structure-Based Computational Selection Compresses Predicted Secondary-Structure Distributions

The iterative computational-selection analysis showed that all three seed backgrounds became strongly enriched for sequences with lower predicted MFE values. This outcome is expected because MFE itself was the selection objective. The important comparative result is therefore not that low-MFE sequences increased, but that the three seed backgrounds differed in their trajectories and relative rankings under identical computational-selection conditions.

This procedure should not be interpreted as SELEX. Experimental SELEX involves enrichment for interaction with a ligand or molecular target, whereas the present algorithm retained sequences solely according to predicted MFE. The term iterative structure-based computational selection more accurately describes the method.

The results show that repeated MFE-based selection can reduce initial differences among mutational neighborhoods, although finer rankings between Sets B and C depended partly on mutation rate. These observations relate to iterative structure-based computational selection under a defined objective function, not to experimentally demonstrated ligand selection or biochemical adaptation.

### Starting-Sequence Composition Influences, But Does Not Fully Explain, the Predicted Landscapes

The three starting sequences differ in nucleotide composition. Set A contains the highest proportion of G and the highest GC content, whereas Sets B and C contain progressively fewer native G residues. However, the mutation rule forces every position away from its native nucleotide. Consequently, a G-rich starting sequence does not generate a G-rich fully mutated library; instead, G is excluded from more positions.

Set A, which contains G at 10 of 20 native positions, had an expected G frequency of only 16.7% in its fully mutated pool. The corresponding expected G frequencies were 20.0% for Set B and 21.7% for Set C. Empirical pool compositions matched these expectations. This feature of the design is important because it means that the observed retained-G advantage cannot be interpreted simply as a consequence of bulk G enrichment in the mutational pools. Nevertheless, starting composition determines which bases are excluded at each position and therefore remains a mechanistic property of each local sequence space. With only three primary seed sequences, the study does not support a general correlation between GC content and predicted foldability. Composition should therefore be discussed as a covariate of the design rather than as an independently established causal explanation.

### G·U Wobble Pairing Affects Effect Magnitude But Does Not Account for Positional Specificity

The default ViennaRNA model permits G·U wobble pairing. The --noGU sensitivity analysis confirms that G·U pairing contributes appreciably to the absolute magnitude of the retained-G effect. When G·U pairing was disabled, mean predicted foldability for retained-G sub libraries fell from 24.84% to 16.56%. Smaller declines also occurred for retained C, U, and A. However, the hierarchy G > C > U > A remained unchanged.

The same strongest position remained identifiable in each seed under the no-G·U model:

- Set A position 16;
- Set B position 5;
- Set C position 17.

Each anchor also continued to outperform the other G-retained positions within the same seed.

At the whole-pool level, disabling G·U pairing reduced predicted foldability for all three seeds but preserved the original order Set B > Set C > Set A.

These findings support a qualified interpretation of the retained G-effect. G·U pairing enhances the predicted stabilizing effect associated with retained G, but it does not fully explain the comparative ranking of the three baseline seed neighborhoods, the identity of the strongest retained position in each seed, or the superiority of each anchor over other G-retained positions within the same sequence. The guanine-associated effect therefore appears to reflect both the thermodynamic advantages available to G-containing secondary structures and position-specific sequence context.

### Cooperative Retained-Position Sets are Statistically Super-Additive But Not Uniformly Compact

The higher-order retention analysis showed that combinations of retained positions produced predicted-foldability values greater than the linear-additive expectation derived from their single-position effects. This super-additive behavior persisted when G·U pairing was disabled, indicating that it was not dependent solely on wobble-pair stabilization.

The term cooperative retained-position set is used here operationally to describe this statistical result. Super-additivity does not by itself establish that all retained positions contact one another directly or occupy a single compact physical core.

The structural-proximity analysis provided partial support for local organization. Some retained-position pairs frequently occurred in the same predicted stem or formed direct predicted base pairs. For example, Set C positions 3 and 4 occurred in the same predicted stem in 67.8% of sequences. Other retained-position pairs were separated in a substantial fraction of the modeled structures.

The best interpretation is therefore that the retained positions act cooperatively at the level of predicted ensemble foldability, while their physical arrangement varies among sequences. Some subsets cluster within common helices or adjacent elements, but the five-position sets do not behave as invariant, structurally monolithic cores.

### Biological Controls Show That Starting-Seed Structure Does Not Predict Neighborhood Richness

The six biological control sequences began from unmutated 20-nt states lacking stable predicted secondary structure under the same RNAfold conditions. Nevertheless, their fully mutated neighborhoods showed greater predicted foldability than those of the three ribosome-derived seeds.

This result does not imply that the control sequences are biologically superior, evolutionarily younger, or more likely to possess biochemical function. Instead, it demonstrates that predicted structure in an individual starting sequence and predicted foldability across its mutational neighborhood are distinct properties.

The result also reinforces the need for caution when interpreting MFE-based accessibility as function. A mutational neighborhood can contain many sequences with favorable predicted secondary-structure energies without being enriched for a particular ligand-binding, catalytic, or biological activity. Rfam (Ontiveros-Palacios et al. 2025) matches were rare among the screened subsets from both ribosome-core-inspired and control libraries, and predicted foldability should not be treated as a proxy for biochemical function.

## Limitations

Several model limitations are central to interpreting the results. First, RNAfold predicts minimum-free-energy secondary structures under a nearest-neighbor thermodynamic model. The calculations used ViennaRNA 2.7.2 with bundled Turner 2004 parameters at 37 °C. No custom salt condition was specified, so the calculations used the program’s default monovalent-salt setting of 1.021 M. This condition does not reproduce physiological intracellular ionic strength or the chemically heterogeneous environment of intact ribosomal RNA.

Second, the model permits G·U wobble pairs by default. The sensitivity analysis shows that these pairs contribute substantially to absolute predicted foldability, especially in G-retained libraries, although the main positional and seed-ranking results remain under --noGU. Third, G-quadruplex prediction was not enabled. Therefore, G-rich sequences were assessed only through the canonical and G·U secondary-structure states represented in the standard RNAfold recursion. G-quadruplexes were not modeled as competing structures. Fourth, standard RNAfold does not model pseudoknots, and the present analysis did not include tertiary interactions, divalent-ion-specific folding, molecular crowding, proteins, ligands, or interactions with surrounding ribosomal RNA. Finally, an MFE structure is a model-selected lowest-energy state, not proof that the RNA predominantly occupies that conformation. The analyses therefore compare relative predictions under a uniform model and should not be interpreted as direct evidence of experimentally populated folds.

Accordingly, predicted foldability and predicted stability refer only to MFE-based secondary-structure behavior under the stated computational conditions and should not be interpreted as evidence of experimentally populated structures, tertiary folding, biochemical activity, or biological function.

## Conclusion

Taken together, the results show that isolated 20-nt seed sequences can define reproducibly different local distributions of predicted secondary-structure stability under a common mutation and thermodynamic-prediction framework. These differences reflect starting-sequence composition, position-specific nucleotide retention, G·U wobble pairing, and super-additive effects among retained-position combinations.

The strongest conclusions are computational: the local sequence neighborhoods are non-equivalent under the ViennaRNA model, the comparative seed ranking is robust to stricter MFE thresholds and exclusion of G·U pairing, and specific retained positions exert effects beyond retained-base identity alone. The study does not establish tertiary structures, ligand binding, catalytic activity, or evolutionary fitness. Experimental structural and functional analyses would be needed to determine whether the computationally identified sequences and positional effects correspond to populated conformations or biochemical phenotypes.

## Data Availability

No datasets were generated or analysed during the current study.
